# Partial Replacement of Fish Meal with Fish Scale Meal Enhances Body Color Brightness and Iridophore Development in Koi (*Cyprinus carpio* var. koi)

**DOI:** 10.3390/ani16142235

**Published:** 2026-07-19

**Authors:** Shengyu Gao, Xiaolong Xu, Tong Ding, Yunxin Cheng, Jiayue Zhang, Huiyi Zheng, Zuoqin Zhou, En Liu, Gaoxiao Xu, Chaofan He

**Affiliations:** 1 Rural Revitalization Collaborative Technology Service Center of Anhui Province, Fuyang Normal University, Fuyang 236041, China; 2024221307@stu.fynu.edu.cn (S.G.); 15551603086@163.com (X.X.); 17355618651@163.com (T.D.); 19155669120@163.com (Y.C.); 15555856827@163.com (J.Z.); 18260442722@163.com (H.Z.); 201808004@fynu.edu.cn (Z.Z.); liuenvip@fynu.edu.cn (E.L.); 2 Modern Industry College of Life Health and Green Food, Fuyang Normal University, Fuyang 236041, China

**Keywords:** ornamental fish, iridophores, brightness, guanine

## Abstract

Body color brightness is a key determinant of ornamental value in koi, yet nutritional strategies targeting structural coloration mediated by iridophores remain underexplored. Fish scale meal (FSM), an abundant fishery by-product, contains guanine and collagen, which may contribute to structural coloration and body brightness. This study evaluated the effects of partial replacement of fish meal with FSM on growth performance, body color parameters, pigment accumulation, iridophore density, and iridophore-related gene expression in koi. A total of 384 koi with an initial body weight of 19.02 ± 0.18 g were randomly assigned to six dietary treatments for 8 weeks, with FSM inclusion levels ranging from 0% to 9.55%, corresponding to 0–100% replacement of dietary fish meal. Growth performance, including final body weight, weight gain rate, and specific growth rate, decreased with increasing FSM inclusion. Body lightness (L*) and scale guanine content increased progressively with FSM level and reached their highest values at 9.55% FSM, whereas redness (a*), yellowness (b*), and total carotenoid content showed the opposite trend. Iridophore density was significantly higher in the 3.82–5.73% FSM groups than in the other treatments. The expression of iridophore-related genes, including *sox10*, *pax3*, *ltk*, *alx4a*, and *foxd3*, showed non-linear responses and generally reached higher levels at moderate FSM inclusion, whereas *pka* showed an opposite transcript pattern. Overall, dietary FSM inclusion elicited trait-dependent responses in koi. Low or no FSM inclusion was preferable for maintaining growth, intermediate inclusion was more favorable for iridophore development, whereas higher FSM inclusion enhanced brightness-related traits. These findings suggest that FSM should be used according to the intended production objective, with inclusion levels adjusted for growth maintenance, iridophore-related coloration improvement, or brightness enhancement.

## Simple Summary

Koi are highly valued ornamental fish, and their commercial value is closely associated with body color quality, including brightness, luster, and vividness. Although nutritional strategies for enhancing red and yellow pigmentation have been widely investigated, dietary regulation of structural coloration, especially iridophore-mediated brightness, remains less understood. Fish scales are abundant fish processing by-products containing collagen and guanine, the latter being involved in the formation of reflective crystals in iridophores. In this study, fish scale meal (FSM) was evaluated as a partial fish meal substitute and potential functional ingredient in koi diets. Its effects on growth performance, body color, guanine deposition, iridophore development, and related gene expression were systematically assessed. The results indicate that FSM affected both nutritional performance and structural coloration traits, suggesting that fish scale-derived ingredients may contribute not only as alternative protein sources but also as functional components for improving ornamental characteristics. This study provides new insight into the nutritional regulation of brightness-related coloration in koi and supports the value-added utilization of fish processing by-products in ornamental aquaculture.

## 1. Introduction

Feed costs account for approximately 40–75% of total aquaculture production expenses, representing a major economic constraint for the industry [1]. Fish meal has traditionally been the primary protein source in commercial aquafeeds due to its balanced amino acid profile and high digestibility [2]. However, increasing global aquaculture demand, combined with limited marine fishery resources, fluctuations in capture fisheries, and strict fishing quotas, has affected the long-term availability and price stability of fish meal in recent years [3,4]. According to recent industry data, global fish meal production was estimated at approximately 5.6 million tons in 2025 [5], while commodity price data showed marked variability in global fish meal prices, as illustrated by an increase from approximately 1992.00 USD/mt in March 2026 to 2423.34 USD/mt in June 2026 [6]. Recent studies have further emphasized that the use of fish meal and fish oil in aquafeeds should be evaluated not only from the perspective of nutritional value, but also in terms of raw material origin, dependence on wild fish resources, price volatility, and broader sustainability concerns [4,7]. Therefore, identifying sustainable and cost-effective alternative protein sources has become a critical research priority.

Fishery by-products, such as fish scales, are often discarded as low-value waste despite their considerable nutritional potential [8]. Improving the utilization of fish processing by-products is considered an important strategy for reducing waste, increasing resource efficiency, and decreasing dependence on conventional marine ingredients [7]. For example, fish scales of common carp (*Cyprinus carpio*) contain more than 60% crude protein and are rich in collagen, lecithin, and chitin [9]. Recent circular economy studies have also highlighted that fish scale waste contains valuable biomaterials, including collagen, hydroxyapatite, chitin, and gelatin, and has potential for conversion into value-added products [10]. However, due to their imbalanced amino acid composition and relatively low digestibility, fish scale-derived ingredients are generally considered as partial protein substitutes or functional feed additives rather than complete replacements for fish meal, particularly when growth performance must be maintained. Therefore, evaluating the appropriate inclusion level of fish scale meal (FSM) is necessary to clarify its potential value in aquafeeds.

Beyond nutritional value, fish scale-derived ingredients may also exert functional effects on fish pigmentation. Fish coloration is determined by both pigmentary and structural mechanisms. Carotenoid-based chromatophores, such as xanthophores and erythrophores, mainly contribute to yellow, orange, and red hues through pigment deposition, whereas iridophores are structural chromatophores that contain reflective guanine crystals rather than carotenoid pigments [11]. The silvery-white coloration of fish scales is primarily attributed to light reflection from multilayered guanine crystals located within iridophore cells [12,13]. In koi, enhanced silver reflectance is closely associated with the arrangement of guanine crystal/cytoplasm stacks and their orientation relative to the scale surface [13,14]. The ordered organization of guanine crystal platelets can reflect incident light and generate brightness, silvery-white appearance, and metallic sheen. Therefore, iridophore-mediated structural coloration differs from carotenoid-based pigmentation and may contribute particularly to body brightness and luster. Because skin pigmentation and brightness are important quality traits affecting the ornamental value of cultured fish [15], improving structural coloration is particularly relevant for ornamental koi. Dietary inclusion of fish scale meal (FSM) may therefore provide not only structural components but also guanine or purine-related precursors that may contribute to intracellular guanine deposition in iridophores. Consequently, valorization of fishery by-products as functional feed ingredients represents a promising strategy to reduce feed costs while improving ornamental traits in aquaculture species.

Koi (*Cyprinus carpio* var. koi) are globally important ornamental fish, and their commercial value is largely determined by body coloration quality. However, dull coloration and low color saturation significantly reduce their market value and aesthetic appeal. In recent years, nutritional strategies aimed at improving koi coloration have primarily focused on carotenoid and astaxanthin supplementation to enhance red and yellow pigmentation [16,17]. Unlike these pigment-based approaches, the present study focused on guanine-based structural brightness and iridophore-related responses, which represent a different coloration mechanism from carotenoid deposition. With the expansion of fish processing and seafood canning industries, fish scales are generated through concentrated processing streams and are often regarded as low-value by-products, providing practical opportunities for collection, reuse, and value-added conversion [10]. Therefore, evaluating FSM as a fishery by-product-derived ingredient links brightness-related coloration improvement with the valorization of underutilized fishery by-products. By comparison, relatively limited attention has been paid to the effects of fishery by-product-derived feed ingredients on iridophore-mediated structural coloration in ornamental fish, as well as to the expression patterns of genes associated with iridophore differentiation and guanine deposition. Therefore, the present study aimed to evaluate the effects of graded dietary FSM inclusion on growth performance, body color parameters, pigment-related biochemical indices, iridophore density, and the expression of genes associated with iridophore development and guanine deposition in koi. By integrating phenotypic, biochemical, histological, and RT-qPCR-based gene expression analyses, this study sought to provide a multifaceted assessment of the associations between dietary FSM inclusion and coloration-related responses in koi.

## 2. Materials and Methods

### 2.1. Feed

A basal diet containing 10% fish meal was formulated. Fish meal was partially replaced by fish scale meal (FSM) at graded levels of 0%, 1.91%, 3.82%, 5.73%, 7.64%, and 9.55%, corresponding to 0–100% replacement of dietary fish meal under an isonitrogenous substitution design. Accordingly, for readability, the dietary treatments were abbreviated as FSM0, FSM2, FSM4, FSM6, FSM8, and FSM10 based on the approximate FSM inclusion levels, corresponding to the actual FSM inclusion levels of 0%, 1.91%, 3.82%, 5.73%, 7.64%, and 9.55%, respectively. The actual FSM inclusion levels are provided in the diet formulation table.

Fish scales used for FSM preparation were obtained from apparently healthy common carp (*Cyprinus carpio*) collected from the Nansi Lake Modern Agricultural Industrial Park, Weishan Lake, Jining, Shandong Province, China. Common carp scales were selected because proximate composition analysis showed that their crude protein content was comparable to that of fish meal, which helped minimize changes to the basal diet formulation when fish meal was replaced by graded levels of FSM under an isonitrogenous substitution design. After collection, visible residual tissues and impurities were manually removed, and the scales were thoroughly rinsed with clean water. The cleaned scales were then dried to constant weight, ground into meal, passed through an 80-mesh sieve, sealed in clean bags, and stored at −20 °C until diet preparation.

The nutritional composition of FSM and the finished experimental diets was analyzed on an air-dry basis. Prior to analysis, FSM and diet samples were ground into fine powder and thoroughly mixed. Crude protein content was determined using the Kjeldahl method, and crude protein was calculated as total nitrogen × 6.25. Ether extract was determined by solvent extraction, and crude ash was measured by incineration in a muffle furnace at 550 °C to constant weight. Calcium content was determined after acid digestion using the calcium oxalate–potassium permanganate titration method, and total phosphorus content was determined using the ammonium molybdate spectrophotometric method.

Collagen type I (Col I) content was determined using a commercial Col I assay kit (H142-1-1; Nanjing Jiancheng Bioengineering Institute, Nanjing, China) according to the manufacturer’s instructions. The absorbance was measured at 450 nm, and Col I concentrations were calculated using a four-parameter logistic standard curve.

The amino acid composition of FSM and the finished experimental diets was determined after acid hydrolysis using high-performance liquid chromatography with an Agilent InfinityLab Poroshell 120 EC-C18 column (Agilent Technologies, Santa Clara, CA, USA). The nutritional composition and amino acid composition of FSM are presented in Table 1.

To minimize the risk of disease transmission from the source fish to the experimental koi, only scales from apparently healthy source fish were used. In addition, the scales were subjected to repeated washing, drying, grinding, and subsequent feed extrusion at 80 ± 5 °C during diet preparation. The prepared pellets were dried to constant weight and stored at −20 °C until use. The same extrusion conditions were applied to all experimental diets to minimize potential processing-related variation among treatments. Previous studies have shown that guanine and fish scale collagen exhibit relatively high thermal stability [18,19]; therefore, extrusion at 80 ± 5 °C was not expected to cause severe thermal degradation of these components. In addition, the nutritional composition and amino acid composition presented in Table 2 and Table 3 were analyzed from the finished diets after processing, thereby reflecting the actual nutrient composition of the diets fed to koi. The formulation and nutritional composition of the experimental diets are presented in Table 2, and the amino acid composition of the experimental diets is shown in Table 3.

Diets were prepared following the extrusion method described by [20]. Briefly, all dry ingredients were ground and passed through an 80-mesh sieve, then thoroughly mixed using a stepwise mixing procedure. Lipid sources (fish oil and soybean oil) were subsequently added and homogenized. Distilled water (10% of dry ingredient weight) was added to facilitate pellet formation. The mixture was extruded into 2.0 mm sinking pellets using a laboratory granulator (YK-160, Changzhou Lihao Granulating Drying Equipment Co., Ltd., Changzhou, China) at 80 ± 5 °C. Pellets were air-dried at 30 °C to constant weight and stored at −20 °C until use.

### 2.2. Fish Feeding Trial

The feeding trial was conducted at the Fuyang Quanzhou Ornamental Fish Breeding Base (Anhui, China). The experimental koi were obtained from the same breeding base, where the feeding trial was also performed. At the beginning of the trial, the fish were approximately 3 months old. Prior to the experiment, koi were acclimated for 1 week in three indoor recirculating aquaculture tanks (3.5 × 3.5 × 3.0 m) connected to the same recirculating system and fed the basal diet; namely, the FSM0 diet in this study. No synthetic pigments or commercial color-enhancing additives were added to the acclimation diet.

After acclimation, all fish were individually weighed before stocking. A total of 384 healthy fish (initial body weight: 19.02 ± 0.18 g) were randomly distributed into 24 floating net cages (1.0 × 1.0 × 1.0 m) installed in the recirculating system, with 16 fish per cage. The 24 cages were evenly distributed among the three tanks, with eight cages in each tank. All tanks were connected to the same recirculating aquaculture system. Cages were randomly assigned to six dietary treatments with four replicates per treatment. The cage was considered the experimental unit for growth performance and statistical analysis. Fish were hand-fed to apparent satiation three times daily (08:00, 12:00, and 16:00) for 8 weeks. During the 8-week feeding trial, only one fish died, and no apparent clinical signs of infectious disease or disease outbreak were observed.

Water quality was maintained within optimal ranges: temperature, 25 ± 2 °C; dissolved oxygen, 5.0–6.0 mg/L; pH, 7.0–7.4; and total ammonia nitrogen, ≤0.04 mg/L.

### 2.3. Sample Collection

At the end of the 8-week feeding trial, feed consumption (FC) and survival rate (SR) were recorded. Fish were fasted for 24 h prior to sampling. All fish were then individually weighed. Two fish per cage were randomly selected for subsequent analyses and anesthetized with eugenol (≥98% purity; Sigma-Aldrich, St. Louis, MO, USA) at 0.1 g/L [21]. Macroscopic images were captured using a high-definition camera (OVOS, OmniVision Technologies, Shanghai, China), and body length (BL) was measured during imaging. Body color was then analyzed using a Chroma Meter (CR-400, Konica Minolta, Tokyo, Japan) from standardized dorsal scale regions on the left and right sides of the dorsal fin. The measured color parameters included L*, a*, and b* values; in the CIELAB color system, L* represents lightness, a* represents the red–green axis, and b* represents the yellow–blue axis. The instrument was calibrated using a standard white calibration plate before measurement. For each fish, three repeated measurements were taken, and the mean value was used for analysis. All color measurements were performed by the same operator. After color measurement, scales were carefully removed using a sterile scalpel and divided into two subsamples; no scale samples were collected before dietary treatment allocation. One subsample was fixed in 4% paraformaldehyde at 4 °C for iridophore observation, and the other was flash-frozen and stored at −20 °C for carotenoid and guanine analyses. Skin tissues beneath sampled scales were collected on ice and stored at −80 °C for gene expression analysis. Finally, visceral mass was excised and weighed for subsequent analysis.

### 2.4. Ethics Statement

All procedures involving animals were conducted in accordance with the Guide for the Care and Use of Laboratory Animals and were approved by the Institutional Animal Care and Use Committee of Fuyang Normal University, China (No. FYNU2025AC005).

### 2.5. Microscopic Examination of Iridophores

Fixed scale samples were removed from 4% paraformaldehyde and gently rinsed with physiological saline. Scales were mounted on glass slides with the dermal side facing upward to facilitate observation of internally located iridophores. Iridophore morphology was observed and photographed using an upright microscope (Olympus BX53, Olympus Corporation, Tokyo, Japan) with a 20× objective under bright-field illumination. Iridophore density was quantified from standardized scale regions and expressed as cells/mm^2^, calculated from five randomly selected fields per sample. Iridophore density and related parameters were quantified using ImageJ software (version 1.54r; National Institutes of Health, Bethesda, MD, USA).

### 2.6. Determination of Total Carotenoids in Scales

Scales stored at −20 °C were thawed, gently washed with distilled water, and blotted dry with filter paper. All procedures were performed under dim light to prevent pigment degradation. Samples were minced and accurately weighed using an analytical balance. Pigments were extracted with an acetone−petroleum ether mixture (2:1, *v*/*v*) in a 50 °C water bath for 8 h. The extract was centrifuged at 4000 rpm for 10 min, and the supernatant was transferred to a separatory funnel. The solution was repeatedly shaken and washed with 5% sodium sulfate solution until the lower aqueous phase became translucent. The organic phase was dehydrated by passage through a funnel containing 10 g of anhydrous sodium sulfate into a 10 mL volumetric flask. Residual pigments were rinsed from the funnel with small volumes of petroleum ether and combined in the flask. The final volume was adjusted to 5 mL. Absorbance was measured at 450 nm using a spectrophotometer with a 1 cm cuvette, with petroleum ether as the blank. Total carotenoid content was calculated according to the following equation:

$$X/(\mathrm{mg}/100\;g)=\frac{A\times y\times 10^{6}}{A1\%1\mathrm{CM}\times 1000\times g}$$

where X is total carotenoid content; A is the absorbance at 450 nm; y is the volume of extract used; A^1^_%1cm_ is the average absorption coefficient of carotenoids (2500); and g is the sample weight.

### 2.7. Determination of Guanine Content in Scales

Frozen scales stored at −20 °C were rinsed, dried, finely minced, and weighed. Samples were transferred to 50 mL round-bottom flasks and mixed with 10 mL of 70% perchloric acid. All procedures involving perchloric acid were performed in a fume hood with appropriate safety precautions. The mixture was hydrolyzed under reflux in a boiling water bath at 100 °C for 30 min and rapidly quenched in an ice bath. The hydrolysate was neutralized to pH 7.0 with 7 mol/L potassium hydroxide. After removal of potassium perchlorate precipitates by vacuum filtration, the filtrate was adjusted to pH 3.0 with 1 mol/L phosphoric acid and centrifuged at 3600 rpm for 10 min. The supernatant was collected, diluted to 50 mL with ultrapure water, and stored at 4 °C for high-performance liquid chromatography (HPLC) analysis.

The chromatographic conditions were optimized based on previous studies for purine analysis. Guanine was quantified using an Agilent InfinityLab Poroshell 120 EC-C18 column. The mobile phase consisted of 0.02 mol/L potassium dihydrogen phosphate buffer (pH 4.6) and methanol (9:1, *v*/*v*). The chromatographic conditions were as follows: flow rate, 0.6 mL/min; column temperature, 25 °C; and injection volume, 5 μL. Detection was performed using a variable wavelength detector (VWD) at 254 and 260 nm, and quantitative data were acquired at 254 nm.

### 2.8. RT-qPCR Analysis of Iridophore-Related Gene Expression

Based on previous studies, *alk*, *pka*, *foxd3*, *sox10*, *pax3*, *alx4a*, *ltk*, and *pnp4a* were selected as candidate genes associated with iridophore development in koi skin. Skin tissue was therefore used for total RNA extraction.

Approximately 20 mg of skin tissue was placed in a centrifuge tube containing 1 mL of TRIzol reagent (Invitrogen, Carlsbad, CA, USA). Total RNA was extracted using a rapid animal tissue/cell total RNA extraction kit (Servicebio, Wuhan, China). RNA quality was assessed with a Bioanalyzer 2100 (Agilent, Santa Clara, CA, USA), and RNA samples were diluted to a uniform concentration before reverse transcription.

Reverse transcription was performed using a reverse transcription kit (Servicebio, Wuhan, China) to synthesize cDNA from total RNA. The reaction was conducted at 25 °C for 5 min, 42 °C for 25 min, and 85 °C for 5 s. The resulting cDNA samples were stored at −20 °C until analysis.

RT-qPCR was performed using 2× Universal Blue SYBR Green qPCR Master Mix (Servicebio, Wuhan, China). The cycling program consisted of pre-denaturation at 95 °C for 30 s, followed by 40 cycles of denaturation at 95 °C for 15 s, annealing at 60 °C for 10 s, and extension at 72 °C for 30 s. Gene-specific amplification was verified by melting curve analysis.

Primers for *alk*, *pka*, *foxd3*, *sox10*, *pax3*, *alx4a*, *ltk*, and *pnp4a* were designed using Primer Premier 5.0 (Premier Biosoft, Palo Alto, CA, USA) and synthesized by Sangon Biotech (Shanghai) Co., Ltd. (Shanghai, China). *β-actin* and *rps11-like* (40S ribosomal protein S11-like) were used as dual reference genes. Both reference genes were validated for expression stability across experimental groups. As shown in Table 4, only primers with amplification efficiencies greater than 90% were used, and the PCR amplification efficiencies of target and reference genes were approximately equal. Relative mRNA expression was calculated using the 2^−ΔΔCt^ method and normalized to the geometric mean of *β-actin* and *rps11-like*.

### 2.9. Growth Calculations and Statistical Analysis

Growth performance and feed utilization were calculated using the following equations:

$$\mathrm{Weight}\;\mathrm{gain}\;\mathrm{rate}\;(\mathrm{WGR},\;\%)\;=\frac{(\mathrm{final}\;\mathrm{average}\;\mathrm{weight}-\mathrm{initial}\;\mathrm{average}\;\mathrm{weight})\;}{\mathrm{initial}\;\mathrm{average}\;\mathrm{weight}}\times \;100$$

$$Specific\;growth\;rate\;(SGR,\%/day)=\frac{ln\;final\;average\;weight-ln\;initial\;average\;weight}{feeding\;days}\times 100$$

$$Feed\;conversion\;ratio\;(FCR)=\frac{total\;feed\;intake}{total\;weight\;gain}$$

$$\mathrm{Survival}\;\mathrm{rate}\;\left(\mathrm{SR},\;\%\right)=\frac{\mathrm{Final}\;\mathrm{number}\;\mathrm{of}\;\mathrm{surviving}\;\mathrm{fish}\;}{\mathrm{Initial}\;\mathrm{number}\;\mathrm{of}\;\mathrm{fish}}\times \;100$$

Data were tested for normality and homogeneity of variance before statistical analysis. One-way analysis of variance (ANOVA) was performed using SPSS 25.0 (IBM Corp., Armonk, NY, USA). When significant differences were detected among treatments, Tukey’s HSD multiple-comparison test was used to compare means. Linear and quadratic regression analyses were performed using the curve estimation function in SPSS to evaluate the dose–response relationships between dietary FSM inclusion level and the measured variables. Dietary FSM inclusion level was used as the independent variable (X), while growth-related parameters, color parameters (L*, a*, and b*), iridophore density, scale total carotenoid content, scale guanine content, and relative expression levels of the eight target genes were used as dependent variables (Y). The linear curve was fitted using the model Y = aX + b, and the quadratic curve was fitted using the model Y = aX^2^ + bX + c. Statistical significance was set at *p* < 0.05. Data are presented as mean ± SEM (*n* = 4, experimental unit: cage).

## 3. Results

### 3.1. Growth Performance

As shown in Table 5, initial body weight (IBW) did not differ among treatments (*p* > 0.05), and no linear or quadratic effects were detected (*p* > 0.05). FBW showed significant linear and quadratic responses (*p* < 0.05), being highest in FSM0 and significantly greater than FSM6–10 (*p* < 0.05), while no differences were observed between FSM2 and FSM4 (*p* > 0.05).

WGR decreased with increasing FSM level (*p* < 0.05), with significant linear and quadratic trends and a better fit for the quadratic model. WGR was higher in FSM0 than FSM10 (*p* < 0.05), while other groups did not differ significantly (*p* > 0.05).

SGR showed a similar response pattern to WGR and differed significantly among treatments (*p* < 0.05), with significant linear and quadratic responses to dietary FSM inclusion. SGR was highest in FSM0 and lowest in FSM10, and FSM0 was significantly higher than FSM10 (*p* < 0.05). The four intermediate groups did not differ significantly from either FSM0 or FSM10, and no significant differences were observed among these four groups (*p* > 0.05).

FI differed significantly among treatments (*p* < 0.05), showing linear (*p* < 0.05) and quadratic (*p* < 0.05) trends. FI peaked in FSM4, significantly higher than all other groups (*p* < 0.05), whereas FSM10 showed the lowest FI (*p* < 0.05). FSM0 was higher than FSM2–8, which did not differ among themselves (*p* > 0.05).

FCR differed significantly among treatments (*p* < 0.05) and showed a significant quadratic response to dietary FSM inclusion, whereas the linear response was not significant (*p* > 0.05). FCR was lowest in FSM0 and FSM10 and highest in FSM4, indicating that feed utilization was poorer at the moderate FSM inclusion level. FSM4 had a significantly higher FCR than FSM0 and FSM10 (*p* < 0.05), while FSM2, FSM6, and FSM8 did not differ significantly from either the lower-FCR groups or FSM4. No significant difference was observed between FSM0 and FSM10 (*p* > 0.05).

VW did not differ among treatments (*p* > 0.05), although a weak linear trend was observed (*p* < 0.05). BL decreased with FSM level (*p* < 0.05), being higher in FSM0 than FSM4–10 (*p* < 0.05), and higher in FSM2 than FSM8–10 (*p* < 0.05). Survival rate showed no differences among treatments (*p* > 0.05).

### 3.2. Body Color Brightness

**Figure 1:**
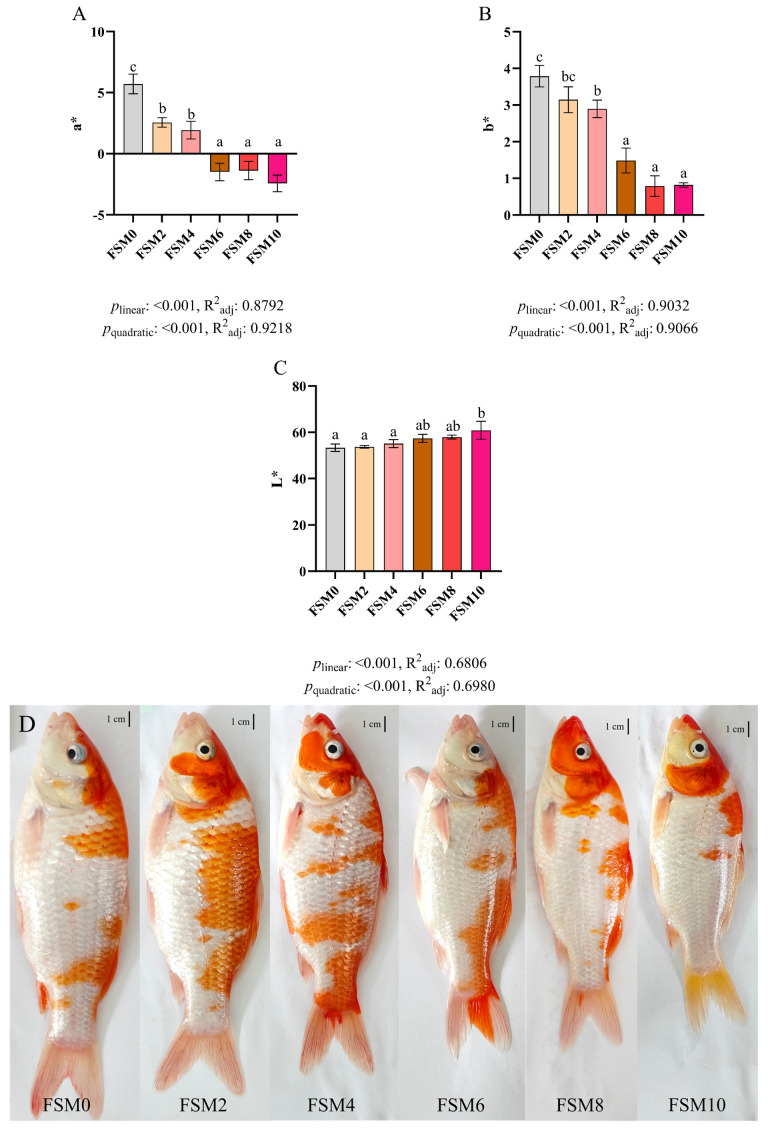
Color parameters and representative body images of koi under different dietary FSM inclusion levels. (**A**) a* value, red–green axis; (**B**) b* value, yellow–blue axis; (**C**) L* value, lightness; (**D**) representative koi images from different dietary treatments. Values are presented as mean ± SEM (*n* = 4). Different lowercase letters indicate significant differences among treatments (*p* < 0.05). Scale bar in panel (**D**) represents 1 cm.

As shown in Figure 1, a*, b*, and L* values showed significant linear and quadratic responses to dietary FSM inclusion (*p* < 0.05). a* and b* values decreased with increasing FSM level, whereas L* increased.

For a*, FSM0 showed significantly higher values than FSM2–10 (*p* < 0.05). FSM2 and FSM4 were significantly higher than FSM6–10 (*p* < 0.05), with no difference between the two former groups (*p* > 0.05). From 5.73% onward, a* values became negative and did not differ among FSM6–10 (*p* > 0.05).

For b*, FSM0 showed significantly higher values than FSM4–10 (*p* < 0.05), but did not differ from FSM2 (*p* > 0.05). FSM4 was significantly higher than FSM6–10 (*p* < 0.05), while no differences were observed among FSM6, FSM8, and FSM10 (*p* > 0.05).

For L*, FSM10 showed the highest value and was significantly higher than FSM0–4 (*p* < 0.05), whereas no differences were observed between FSM6 and FSM8, or between these groups and other intermediate levels (*p* > 0.05).

### 3.3. Iridophore Density

**Figure 2:**
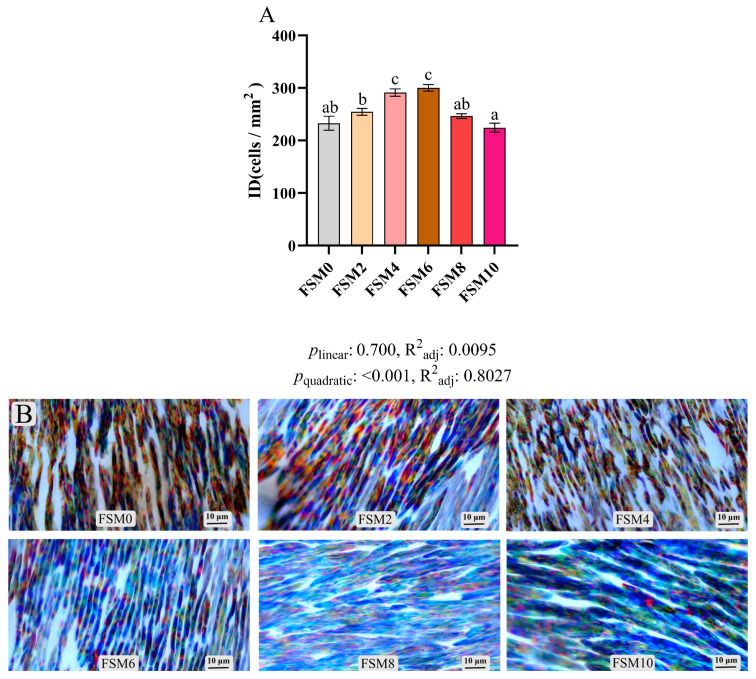
Scale iridophore density and representative microscopic images of scale iridophores in koi. (**A**) Iridophore density (ID, cells/mm^2^); (**B**) representative microscopic images of scale iridophores. Scale bars in panel B represent 10 μm. Values are presented as mean ± SEM (*n* = 4). Different lowercase letters indicate significant differences among treatments (*p* < 0.05). The colors in panel B reflect the optical appearance of iridophores under bright-field microscopy and do not represent staining or pseudo-color labeling.

As shown in Figure 2, iridophore density (ID) showed a significant quadratic response (*p* < 0.05) without a linear effect (*p* > 0.05). ID peaked in FSM4 and FSM6, both significantly higher than other groups (*p* < 0.05). FSM10 showed the lowest ID (*p* < 0.05), while FSM2 was higher than FSM10 (*p* < 0.05). FSM0 and FSM8 showed intermediate values with no differences among groups (*p* > 0.05).

### 3.4. Carotenoids and Guanine

**Figure 3:**
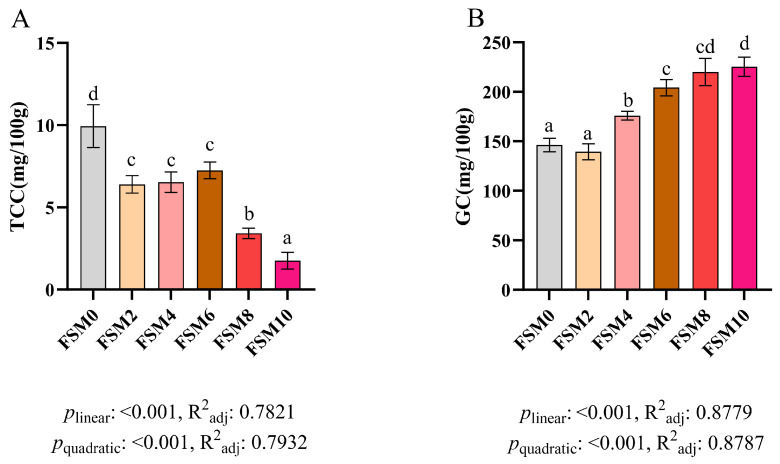
Total carotenoid and guanine contents in koi scales under different dietary FSM inclusion levels. (**A**) Total carotenoid content (TCC); (**B**) guanine content (GC). Values are presented as mean ± SEM (*n* = 4). Different lowercase letters indicate significant differences among treatments (*p* < 0.05).

As shown in Figure 3, total carotenoid content (TCC) decreased significantly with FSM level (*p* < 0.05), while guanine content (GC) increased (*p* < 0.05). Both showed significant linear and quadratic relationships (*p* < 0.05).

TCC was highest in FSM0 and significantly higher than all other groups (*p* < 0.05). FSM2–6 were intermediate and higher than FSM8–10 (*p* < 0.05), while FSM10 was the lowest (*p* < 0.05).

GC was lowest in FSM0–2 (*p* < 0.05), intermediate in FSM4 (*p* < 0.05), and highest in FSM6–10 (*p* < 0.05), with FSM10 showing the maximum value (*p* < 0.05).

### 3.5. Expression of Genes Related to Iridophore Development

**Figure 4:**
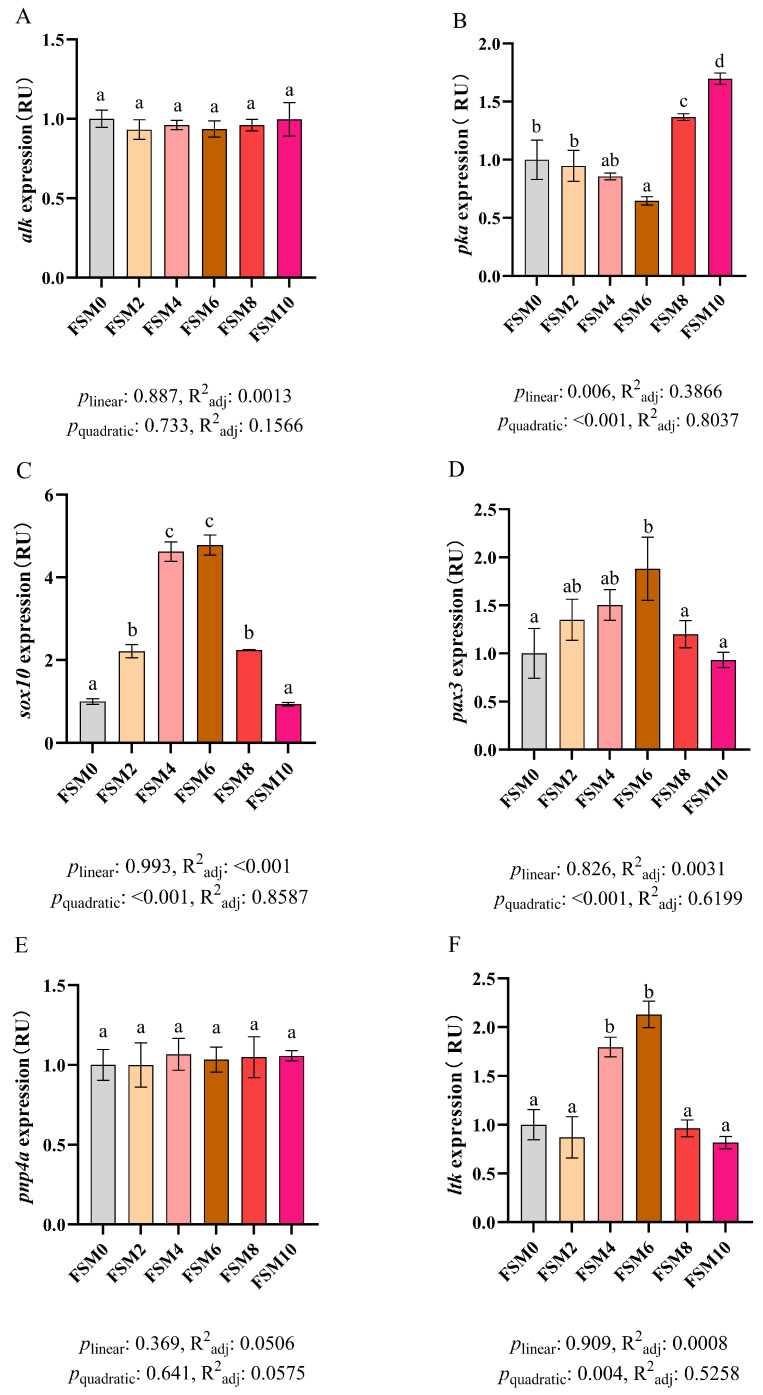

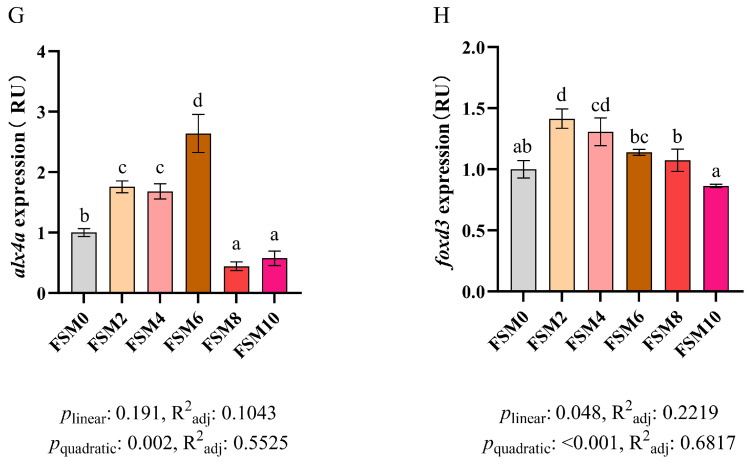
Relative mRNA expression levels of genes related to iridophore development in koi skin. Gene abbreviations: (**A**) *alk*, ALK receptor tyrosine kinase; (**B**) *pka*, regulatory subunit of type II PKA R-subunit domain containing 1; (**C**) *sox10*, transcription factor Sox-10; (**D**) *pax3*, paired box 3; (**E**) *pnp4a*, purine nucleoside phosphorylase 4a; (**F**) *ltk*, leukocyte receptor tyrosine kinase; (**G**) *alx4a*, ALX homeobox 4a; (**H**) *foxd3*, forkhead box protein D3. Values are presented as mean ± SEM (*n* = 4). Different lowercase letters indicate significant differences among treatments (*p* < 0.05).

As shown in Figure 4, the relative mRNA expression levels of *alk* and *pnp4a* did not differ significantly among treatments (*p* > 0.05), and no significant linear or quadratic trends were observed (*p* > 0.05).

The relative mRNA expression of *pka* showed significant linear (*p* = 0.006) and quadratic (*p* < 0.001) responses to dietary FSM inclusion, with the lowest expression observed in FSM6 and the highest expression observed in FSM10 (*p* < 0.05). Specifically, *pka* expression in FSM10 was significantly higher than that in all other groups (*p* < 0.05), whereas FSM6 showed the lowest expression level and was significantly lower than FSM0, FSM2, FSM8, and FSM10 (*p* < 0.05). In addition, *pka* expression in FSM8 was significantly higher than that in FSM0, FSM2, and FSM4 (*p* < 0.05).

*sox10*, *ltk*, *pax3*, and *alx4a* all showed significant quadratic responses (*p* < 0.01), with expression generally increasing at moderate FSM levels and decreasing at higher levels.

*sox10* and *ltk* followed similar patterns, peaking at FSM4 and FSM6, which were significantly higher than all other treatments (*p* < 0.05), with no difference between these two peak groups (*p* > 0.05). For *sox10*, FSM0 and FSM10 showed the lowest expression and did not differ from each other (*p* > 0.05), but were significantly lower than intermediate treatments (*p* < 0.05). The FSM2 and FSM8 groups showed intermediate expression with no difference between them (*p* > 0.05). For *ltk*, all groups except FSM4 and FSM6 showed no significant differences among themselves (*p* > 0.05).

*pax3* was highest in FSM6, significantly higher than FSM0, FSM8, and FSM10 (*p* < 0.05), while other groups did not differ significantly (*p* > 0.05).

*alx4a* showed a distinct pattern, with FSM6 significantly higher than all other treatments (*p* < 0.05). FSM2 and FSM4 showed intermediate expression, significantly higher than FSM0, FSM8, and FSM10 (*p* < 0.05), while FSM8 and FSM10 were the lowest and did not differ (*p* > 0.05).

*foxd3* showed significant linear (*p* = 0.048) and quadratic (*p* < 0.001) effects, peaking at FSM2 and reaching its lowest level at FSM10 (*p* < 0.05). FSM2 was significantly higher than FSM0 and FSM6–10 (*p* < 0.05), while FSM4 showed no significant difference from FSM2 (*p* > 0.05). FSM10 was significantly lower than FSM6 and FSM8 (*p* < 0.05) but did not differ from FSM0 (*p* > 0.05).

## 4. Discussion

This study evaluated the feasibility of FSM as a partial substitute for FM in koi diets and demonstrated a trade-off between growth performance and ornamental coloration. Specifically, the increases in L* value and guanine content at higher FSM inclusion levels occurred together with reductions in growth-related performance, indicating that the brightness-enhancing effect of high FSM inclusion was accompanied by a growth cost. Increasing FSM inclusion resulted in a general reduction in FBW, WGR, and SGR, accompanied by a decrease in BL, with more pronounced effects observed at higher substitution levels. These results suggest that high levels of FM replacement with FSM may negatively affect somatic growth, whereas low to moderate inclusion levels appear to be tolerable without significant growth impairment.

The growth depression observed at higher FSM inclusion levels may be associated with changes in dietary nutrient quality and availability rather than crude protein level alone. Although the experimental diets were formulated to be comparable in crude protein content, increased FSM inclusion altered the dietary amino acid composition, collagen content, and mineral composition, as reflected by the nutritional and amino acid composition data presented in Table 2 and Table 3. FSM protein is largely derived from collagen, which differs from fish meal in amino acid composition, particularly in the proportions of some essential amino acids required for protein synthesis [29,30]. In addition, the structural characteristics of fish scales, including collagen and hydroxyapatite, may potentially affect nutrient accessibility and digestive utilization [31,32,33,34]. Moreover, progressive replacement of fish meal by FSM may have reduced the contribution of fish-meal-derived lipid-associated nutrients, such as phospholipids, which are important for lipid transport and metabolic regulation [35,36]. Taken together, the reduced growth performance at high FSM inclusion levels may be partly related to the combined effects of altered amino acid composition, increased collagen and mineral fractions, and reduced contribution of fish-meal-associated functional nutrients. Further studies including digestibility assessment, amino acid availability analysis, and lipid class characterization would help clarify these nutritional responses.

FI showed a non-linear response to FSM inclusion, peaking at moderate levels and decreasing at higher substitution ratios. This pattern may be related to improved palatability at moderate inclusion, potentially due to free amino acids and small peptides generated from collagen hydrolysis, which are known feeding stimulants in fish [37]. It may also reflect a compensatory feeding response to reduced nutrient availability or amino acid imbalance caused by partial fish meal replacement. However, the increased feed intake at moderate FSM inclusion was not accompanied by a proportional increase in growth, resulting in a higher FCR and poorer feed utilization. At the highest FSM inclusion levels, nutritional imbalance may have become more pronounced, and possible reductions in palatability or digestibility, partly associated with increased mineral content and amino acid imbalance [34], may have limited further compensation through increased feed intake. Therefore, FCR should be interpreted together with FI and growth-related indices, because the lower FCR observed at the highest FSM level occurred alongside reduced feed intake and lower growth performance.

Body color is a key determinant of koi ornamental value, and the present results indicate that FSM inclusion markedly influences skin color parameters. Increasing FSM levels led to a decrease in a* and b* values and an increase in L*, suggesting a shift from warm pigmentation toward enhanced brightness and reflectance.

The reduction in redness and yellowness is likely associated with decreased dietary carotenoid availability, as fish meal is an important source of carotenoids and their precursors [38]. This was supported by the observed reduction in total carotenoid content across treatments. A possible explanation is that limited carotenoid supply may alter metabolic allocation toward essential physiological processes, although the precise regulatory mechanisms remain unclear [39].

In contrast, increased brightness (L*) is closely related to guanine-based structural coloration. Guanine crystals within iridophores form multilayer reflectors responsible for light reflection [12]. The highest L* values observed at the highest FSM level suggest enhanced structural reflectance, which is consistent with increased guanine content. Fish scales contain both guanine and collagen [13,40], and collagen-derived glycine could theoretically participate in purine biosynthesis [41]. Thus, FSM-derived guanine-related components and collagen-derived amino acids may provide a plausible explanation for the increased guanine accumulation and enhanced structural coloration observed in this study. Nevertheless, this proposed pathway remains to be verified, because purine metabolites, glycine availability, and enzymes involved in de novo purine synthesis were not directly examined. Further studies integrating purine metabolomics, amino acid availability analysis, and key enzyme assays would help clarify whether FSM-derived components contribute directly or indirectly to guanine deposition in iridophores.

Iridophore density showed a clear quadratic response to FSM inclusion, with maximal values at moderate levels and a decline at higher substitution rates. This suggests that moderate FSM inclusion may provide a more favorable nutritional environment for iridophore development, whereas excessive inclusion may impose physiological constraints [41,42].

At moderate levels, FSM may supply beneficial structural and biochemical components, including collagen, hydroxyapatite, and guanine [33,34,41,42], while residual fish meal continues to provide complementary nutrients such as essential amino acids and lipid-associated cofactors [30]. At higher inclusion levels, the increased mineral fraction of FSM, particularly the elevated dietary calcium level as reflected by the nutritional composition presented in Table 2, may have contributed to mineral-related nutritional imbalance. Previous studies have suggested that high dietary calcium may affect zinc utilization [43], and zinc is required for the function of some zinc-finger transcription factors involved in pigment cell regulation [44]. These mineral-related interactions may be associated with the reduced iridophore density observed at high FSM levels. However, because zinc status, tissue mineral composition, and mineral absorption were not directly determined, further studies are needed to clarify the relationship between dietary mineral balance and iridophore development in koi.

Interestingly, despite the decrease in iridophore density at high substitution levels, L* continued to increase. This suggests that structural coloration is not solely dependent on cell number, but may also be influenced by intracellular organization of guanine crystals. Enhanced substrate availability may potentially influence guanine crystal organization or stacking, thereby increasing reflectance efficiency at the cellular level [12,13]. In the present study, bright-field microscopy was used to quantitatively determine iridophore density, whereas guanine crystal size, arrangement, spacing, and reflectance spectra were not directly measured. Therefore, future studies using higher-resolution imaging and optical approaches, such as reflective light microscopy, polarized light microscopy, electron microscopy, and reflectance spectral analysis, are needed to further characterize iridophore structure and guanine crystal arrangement.

To further examine the transcriptional responses associated with iridophore development, the expression of genes related to neural crest-derived pigment cell development was analyzed. The iridophore-related genes *sox10*, *pax3*, *ltk*, *alx4a*, and *foxd3* showed significant non-linear responses to dietary FSM, generally peaking at moderate inclusion levels, particularly around FSM6. This expression pattern broadly corresponded with the changes in iridophore density, suggesting that moderate FSM inclusion was associated with coordinated transcriptional changes in iridophore-related genes [25].

Functionally, *sox10* and *pax3* are critical regulators of pigment cell progenitor survival and specification [45,46], while *ltk* plays a central role in iridophore lineage commitment [47]. *alx4a* also contributes to pigment cell differentiation processes [25,47]. The increased expression of these genes at moderate FSM levels was consistent with the observed enhancement of iridophore density, suggesting a transcriptional association between these genes and iridophore development. However, their involvement at the pathway level requires further experimental validation.

In contrast, *pka* expression exhibited an opposite trend, reaching its lowest level at moderate FSM inclusion. Since PKA signaling has been associated with inhibitory regulation of iridophore differentiation [23], the reduced *pka* transcript level at moderate FSM inclusion may be related to the observed iridophore-related responses. However, *pka* mRNA abundance alone cannot determine PKA pathway activity, and cAMP concentration, PKA enzymatic activity, phosphorylation of downstream targets, and related signaling markers were not measured in this study. Therefore, the role of PKA signaling in FSM-associated iridophore responses remains to be further validated. Meanwhile, *alk* and *pnp4a* expression remained relatively stable across treatments, suggesting that FSM inclusion was mainly associated with changes in selected iridophore differentiation-related genes rather than broad alterations in neural crest-related gene expression [25,48]. The stability of *pnp4a* may also suggest that endogenous guanine-related synthesis was not strongly altered across treatments, although this possibility requires further validation [49].

Together, these phenotypic, biochemical, and transcriptional results suggest that dietary FSM inclusion was associated with changes in both carotenoid-related pigmentation and guanine-based structural coloration. At low inclusion levels, carotenoid deposition predominates, resulting in stronger warm coloration. At moderate levels, a balance between carotenoid reduction and guanine accumulation leads to improved brightness with retained pigmentation. At high inclusion levels, carotenoid depletion becomes dominant, while structural reflectance is maximized, resulting in a silvery-white appearance. Because scale samples were collected only at the end of the feeding trial, baseline scale pigment and guanine status before dietary treatment could not be directly compared among groups.

Because different endpoints showed distinct dose–response patterns, a single universal optimal FSM inclusion level should not be inferred across all traits. Growth performance and red/yellow coloration-related traits, including WGR, BL, a*, b*, and TCC, generally showed linear decreases with increasing FSM inclusion, indicating that no or lower FSM inclusion was more favorable for maintaining growth and red/yellow coloration. In contrast, brightness-related endpoints, including L* and guanine content, increased with dietary FSM inclusion and reached the highest observed values at the highest tested level, suggesting that high FSM inclusion enhanced brightness-related structural coloration within the tested range. However, this effect should be interpreted as a brightness-specific response rather than a general benefit of high-level FSM replacement. Moreover, iridophore density and most iridophore-related genes, including *sox10*, *pax3*, *alx4a*, and *ltk*, showed non-linear or quadratic responses, with higher values mainly observed at moderate FSM inclusion levels, especially around FSM6. These findings indicate that the response to dietary FSM inclusion was endpoint-dependent, with growth, brightness, and iridophore-related traits showing different favorable response ranges within the tested inclusion gradient. Nevertheless, the practical interpretation of FSM inclusion levels should also consider the biological source of FSM. The use of by-products derived from the same or closely related species should be carefully considered from ethical, biosafety, regulatory, and practical application perspectives. Therefore, the present study should be regarded as a controlled experimental evaluation of the functional potential of FSM rather than a direct recommendation for unrestricted same-species recycling in commercial feed production.

Beyond its biological effects, the practical value of FSM also depends on whether it can be obtained and processed at a scale and cost acceptable for feed production. The expanding fish processing and seafood canning industries generate considerable amounts of low-value by-products, including fish scales, which are often underutilized despite their potential economic value [10]. Based on previous studies as well as its nutritional composition and amino acid profile, FSM may have potential to be developed into value-added products or animal feed supplements [9,10], although limitations such as amino acid imbalance should still be considered. In the present study, moderate FSM inclusion improved body color brightness without causing severe growth impairment, suggesting its potential application value in koi feed. However, guanine availability, collagen digestibility, and pellet physical stability were not directly determined in the present study, as the primary focus was to evaluate the effects of FSM on body color brightness and iridophore-related responses in koi. Therefore, future studies should further assess pellet stability together with the digestibility and bioavailability of guanine- and collagen-related components in FSM-based diets. In addition, large-scale industrial application would require stable systems for fish scale collection, processing, storage, and quality control. Further pilot-scale production trials and techno-economic assessments are needed to evaluate the economic feasibility, supply stability, biosafety, and quality consistency of FSM before commercial application.

## 5. Conclusions

Partial replacement of fish meal with FSM produced endpoint-specific effects on growth performance, body color parameters, guanine accumulation, and iridophore development in koi. Growth-related performance was more favorable in the control or lower-FSM groups, indicating that low or no FSM inclusion was preferable for maintaining growth-related performance. In contrast, moderate FSM inclusion levels, particularly FSM6, corresponding to an actual FSM inclusion level of 5.73%, promoted iridophore development, guanine accumulation, and body color brightness while maintaining acceptable growth performance. Higher FSM inclusion levels enhanced brightness-related responses but tended to reduce growth performance and did not further increase iridophore density.

These findings indicate that FSM can be used as a functional ingredient derived from fish processing by-products in koi feed. However, a clear trade-off was observed between brightness enhancement and growth performance with increasing FSM inclusion: higher FSM levels enhanced brightness-related responses, whereas growth performance tended to decline. Thus, high FSM inclusion may be useful when brightness enhancement is prioritized, but it should not be regarded as a universally optimal replacement level. Therefore, the practical inclusion level of FSM should be adjusted according to the target production objective, such as growth maintenance, iridophore-related coloration improvement, or brightness enhancement. This study provides a potential strategy for the valorization of fish processing by-products and the development of functional feeds for ornamental fish.

## Figures and Tables

**Table 1:** Nutritional composition and amino acid composition of FSM on an air-dry basis.

Nutritional Composition			
Item	Content (%)	Item	Content (%)
Crude protein	63.21	Collagen	48.71
Ether extract	0.53	Calcium	13.21
Crude ash	33.05	Total phosphorus	6.13
Essential amino acids		Non-essential amino acids	
Item	Content (%)	Item	Content (%)
Lysine	4.45	Aspartic acid	3.64
Methionine	1.76	Tyrosine	1.96
Isoleucine	1.99	Serine	2.46
Leucine	1.25	Glutamic acid	6.94
Phenylalanine	1.26	Proline	5.81
Valine	2.02	Glycine	16.50
Histidine	1.72	Alanine	6.02
Arginine	1.63	Cystine	1.55
Threonine	0.61		

**Table 2:** The formulation and approximate composition of the experimental diet.

Ingredients (%)	FSM0	FSM2	FSM4	FSM6	FSM8	FSM10
Fish meal	10.00	8.00	6.00	4.00	2.00	0.00
Soybean meal	24.00	24.00	24.00	24.00	24.00	24.00
Rapeseed meal	24.00	24.00	24.00	24.00	24.00	24.00
Wheat gluten	12.84	12.84	12.84	12.84	12.84	12.84
Wheat flour	23.31	23.31	23.31	23.31	23.31	23.32
Soybean oil	2.47	2.47	2.47	2.47	2.47	2.47
Fish oil	2.47	2.56	2.65	2.74	2.83	2.92
Ca(H_2_PO_4_)_2_	0.15	0.15	0.15	0.15	0.15	0.15
Premix ^a^	0.15	0.15	0.15	0.15	0.15	0.15
Choline chloride	0.20	0.20	0.20	0.20	0.20	0.20
Lysine (98.5%)	0.30	0.30	0.30	0.30	0.30	0.30
Methionine	0.10	0.10	0.10	0.10	0.10	0.10
Fish scale meal	0.00	1.91	3.82	5.73	7.64	9.55
Crude protein	39.91	39.89	40.02	39.97	40.23	40.15
Ether extract	6.44	6.52	6.41	6.55	6.60	6.47
Crude ash	4.80	5.17	5.64	5.92	6.29	6.76
Collagen	0.37	1.25	2.05	3.03	3.89	4.72
Calcium	0.68	0.82	1.03	1.28	1.40	1.50
Total phosphorus	0.75	0.85	0.89	0.97	1.04	1.10
Dietary guanine content (mg/100 g)	42.08	44.47	44.68	47.45	52.24	57.20

Note: ^a^ Vitamin premix (mg/kg diet): vitamin B_1_, 12 mg; vitamin B_2_, 5 mg; vitamin B_5_, 30 mg; vitamin B_6_, 6 mg; vitamin B_12_, 0.05 mg; vitamin D_3_, 5 mg; vitamin E, 40 mg; vitamin K_3_, 5 mg; inositol, 100 mg; niacin, 35 mg; folic acid, 2 mg, biotin, 0.05 mg; retinol acetate, 25 mg; ascorbic acid, 500 mg; ethoxyquin, 150 mg; corn protein meal, 585 mg; KCl, 10 mg; KI (1%), 3 mg; CoCl_2_·6H_2_O (1%), 0.35 mg; CuSO_4_·5H_2_O, 0.7 mg; FeSO_4_·H_2_O, 20 mg; ZnSO_4_·H_2_O, 10 mg; MnSO_4_·H_2_O, 4 mg; Na_2_SeO_3_·5H_2_O (1%), 3.25 mg; MgSO_4_·7H_2_O, 150 mg; Ca(H_2_PO_4_)_2_·H_2_O, 1000 mg; NaCl, 6.8 mg; Zeolite, 292 mg.

**Table 3:** Amino acid composition of the experimental diets on an air-dry basis (g/100 g).

Amino Acids	FSM0	FSM2	FSM4	FSM6	FSM8	FSM10
Essential amino acids						
Lysine	1.84	1.77	1.67	1.60	1.55	1.46
Methionine	0.73	0.74	0.72	0.70	0.70	0.72
Isoleucine	1.53	1.53	1.45	1.42	1.41	1.35
Leucine	2.71	2.81	2.72	2.62	2.61	2.45
Phenylalanine	1.91	1.82	1.79	1.84	1.83	1.73
Valine	1.73	1.78	1.74	1.66	1.66	1.61
Histidine	0.95	0.94	0.92	0.87	0.90	0.87
Arginine	2.21	2.31	2.29	2.33	2.32	2.27
Threonine	0.64	0.65	0.68	0.69	0.7	0.72
Total Essential amino acids	14.21	14.24	13.92	13.72	13.64	13.14
Non-essential amino acids						
Aspartic acid	3.14	3.06	3.02	2.99	2.94	2.95
Tyrosine	1.23	1.26	1.28	1.24	1.22	1.19
Serine	1.89	1.89	1.90	1.87	1.82	1.87
Glutamic acid	8.83	8.63	8.93	8.59	8.86	8.56
Proline	2.70	2.83	2.92	2.97	3.01	3.12
Glycine	1.79	2.05	2.31	2.49	2.78	2.92
Alanine	1.67	1.69	1.79	1.82	1.79	1.88
Cystine	0.67	0.70	0.68	0.72	0.72	0.76
Total Non-essential amino acids	21.92	22.11	22.83	22.69	23.14	23.25

**Table 4:** Real-time PCR primer nucleotide sequence.

Target Gene	Forward (5′–3′)	Reverse (5′–3′)	References
*alk*	CCGCAGTAACCAAGAGGTGT	CAGGCAAAGGCACATTCACC	[21,22]
*pka*	ACGCATAGATGGCGGAGAGA	CTATCCGCGTCTTGACCTTGA	[23]
*sox10*	CTCGCTCCTCACACACAACGAG	TGAATCCATCCGCCGTCTTC	[24]
*pax3*	AAGGGCATTAGCATCCCACAGTAT	ATAGTTGGGTCCTCGGATTGG	[24]
*pnp4a*	TGTAAAACACTGGGCTATTCCAT	ATACAGCCTTCACAGCACAAAA	[23]
*ltk*	ATTTCTGGCATTTGATTCGCTG	ATATTAAGCCCTCATCTTCCCC	[25]
*alx4a*	CGCATCAGGCATGGTAGACTT	GCAGATTGCTCTTGGGGGAC	[26]
*foxd3*	GGCGATGTTACTGTGTTTCCC	GTGGTTTTGCACCGAGGAGT	[23]
*β-actin*	GCTATGTGGCTCTTGACTTCGA	CCGTCAGGCAGCTCATAGCT	[27]
*rps11-like*	GAACGCTTTTAGTTCGGCCC	AAGCCCTCTCGTTCTGTGTG	[28]

Note: *alk*: ALK receptor tyrosine kinase; *pka*: regulatory subunit of type II PKA R-subunit domain containing 1; *sox10*: transcription factor Sox-10; *pax3*: paired box 3; *pnp4a*: purine nucleoside phosphorylase 4a; *ltk*: leukocyte receptor tyrosine kinase; *alx4a*: ALX homeobox 4a; *foxd3*: forkhead box protein D3; *β-actin*: beta-actin (*ACTB*). *rps11-like*: 40S ribosomal protein S11-like.

**Table 5:** Growth performance of koi (*Cyprinus carpio* var. koi) under different fish scale meal replacement levels of fish meal.

Parameters	FSM Level						ANOVA *p* > F	Polynomial Contrasts			
	FSM0	FSM2	FSM4	FSM6	FSM8	FSM10		Linear		Quadratic	
								*p*-Value	R^2^_adj_	*p*-Value	R^2^_adj_
IBW	18.91 ± 0.19 ^a^	18.88 ± 0.19 ^a^	18.96 ± 0.12 ^a^	19.00 ± 0.10 ^a^	19.01 ± 0.10 ^a^	18.99 ± 0.04 ^a^	0.814	0.333	0.059	0.635	0.059
(g)											
FBW	75.57 ± 2.19 ^b^	67.68 ± 1.26 ^ab^	65.26 ± 1.00 ^ab^	63.86 ± 5.22 ^a^	61.41 ± 4.13 ^a^	58.52 ± 6.08 ^a^	0.003	<0.001	0.681	<0.001	0.712
(g)											
WGR	224.78 ± 11.96 ^b^	193.24 ± 16.09 ^ab^	186.28 ± 9.51 ^ab^	180.39 ± 15.36 ^ab^	178.22 ± 14.05 ^ab^	154.21 ± 18.98 ^a^	0.002	<0.001	0.662	<0.001	0.673
(%)											
SGR	2.06 ± 0.07 ^a^	1.91 ± 0.11 ^ab^	1.88 ± 0.06 ^ab^	1.84 ± 0.10 ^ab^	1.83 ± 0.09 ^ab^	1.79 ± 0.09 ^b^	0.039	0.001	0.484	0.002	0.511
%/day											
FI	58.58 ± 0.91 ^c^	55.20 ± 0.60 ^bc^	64.77 ± 1.79 ^d^	57.17 ± 2.31 ^bc^	53.73 ± 1.05 ^ab^	50.00 ± 2.34 ^a^	<0.001	0.015	0.317	0.002	0.572
(g)											
FCR	1.45 ± 0.08 ^a^	1.59 ± 0.01 ^ab^	1.80 ± 0.13 ^b^	1.64 ± 0.08 ^ab^	1.59 ± 0.12 ^ab^	1.51 ± 0.12 ^a^	0.007	0.867	0.002	0.003	0.553

VW	5.27 ± 0.31 ^a^	5.47 ± 0.40 ^a^	5.63 ± 0.40 ^a^	5.67 ± 0.45 ^a^	5.70 ± 0.53 ^a^	5.47 ± 0.60 ^a^	0.85	0.044	0.405	0.132	0.345
(g)											
BL	26.07 ± 0.97 ^c^	24.50 ± 1.67 ^bc^	22.67 ± 0.83 ^ab^	21.27 ± 0.83 ^ab^	21.27 ± 0.87 ^a^	21.50 ± 0.50 ^a^	<0.001	<0.001	0.651	<0.001	0.776
(cm)											
SR	97.92 ± 3.61 ^a^	100 ± 0.00 ^a^	100 ± 0.00 ^a^	100 ± 0.00 ^a^	100 ± 0.00 ^a^	100 ± 0.00 ^a^	0.458	0.148	0.126	0.139	0.231
(%)											

Note: IBW, Initial body weight; FBW, Final body weight; WGR, Weight gain rate; SGR, Specific Growth Rate; FI, Feed intake; FCR, Feed Conversion Ratio; VW, Visceral Weight; BL, Body Length; SR, Survival Rate. Values with different superscript letters within the same row differ significantly (*p* < 0.05).

## Data Availability

The data presented in this study are available within the manuscript.
